# A Retroperitoneal Calcified Mass: Synovial Sarcoma

**DOI:** 10.7759/cureus.35999

**Published:** 2023-03-10

**Authors:** Alper Gok, Görkem Özenç, Serafettin Kaymak, Azmi Levent Sagnak, Muhammet Abdurrahim Imamoglu

**Affiliations:** 1 Department of Urology, University of Health Sciences, Diskapi Yildirim Beyazit Training and Research Hospital, Ankara, TUR; 2 Department of Urology, Haymana State Hospital, Ankara, TUR

**Keywords:** retroperitoneal tumors, therapeutic management, synovial sarcoma, retroperitoneal neoplasms, retroperitoneal

## Abstract

Retroperitoneal synovial sarcoma is an extremely rare and aggressive tumor. Although it does not have a typical radiological appearance, it can sometimes appear as a calcified mass. The most important step in the treatment of synovial sarcoma is complete resection. However, despite complete resection, local recurrence and systemic spread rates remain high, and chemo/radiotherapy may be considered in high-risk patients.

## Introduction

Retroperitoneal masses (RPMs) are rare lesions. Although they are frequently primary malignant lesions, they can also present as benign or metastases of another region, although this is not as common [1,2]. In addition to sarcomas, the differential diagnoses of RPMs include schwannomas, paragangliomas, lymphomas, germ cell tumors, testicular tumor metastases, and desmoid tumors. Soft-tissue sarcomas (STSs) are mostly localized in the extremities, but approximately 15% are located in the retroperitoneum. These tumors account for approximately one-third of all RPMs [1]. The annual incidence of retroperitoneal sarcomas (RPSs) is approximately 2.7/106 [3]. The most common histological subgroups of these rare tumors of mesenchymal origin are liposarcomas, leiomyosarcomas, and malignant fibrous histiocytomas. Synovial sarcomas (SSs), which are generally seen in young people, constitute approximately 5-10% of all STSs, representing the fourth most common subgroup of STSs.

Because RPMs do not usually cause symptoms due to the expandable nature of the retroperitoneum, most have large dimensions at the time of first diagnosis. For example, in a previous study, it was stated that 60% of RPSs were >10 cm and 85% were >5 cm at the time of diagnosis [1]. Although they do not have specific findings defined by radiological methods, some have been reported to contain calcified foci on computed tomography (CT) [4]. The main step in the treatment is the removal of the mass with a wide excision. However, this is often difficult or sometimes impossible due to the deep location of RPS and its ability to invade surrounding vital tissues and organs (aorta, vena cava, diaphragm, intestines, and neural structures). Although RPSs are aggressive tumors, they are unlikely to be metastatic at the time of first diagnosis. They most commonly metastasize to the lungs and liver, and the overall survival time in metastatic disease is very low (an average of 13 months) [2]. In this paper, we present a case of retroperitoneal SS treated in our clinic due to its rare nature.

## Case presentation

**Figure 1:**
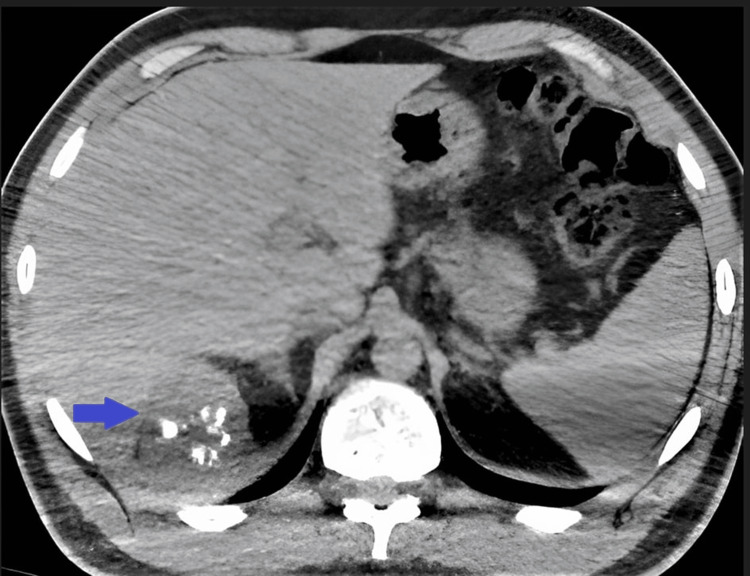
Unenhanced computed tomography image in the axial plane of a centrally calcified retroperitoneal mass localized between the diaphragm and liver.

**Figure 2:**
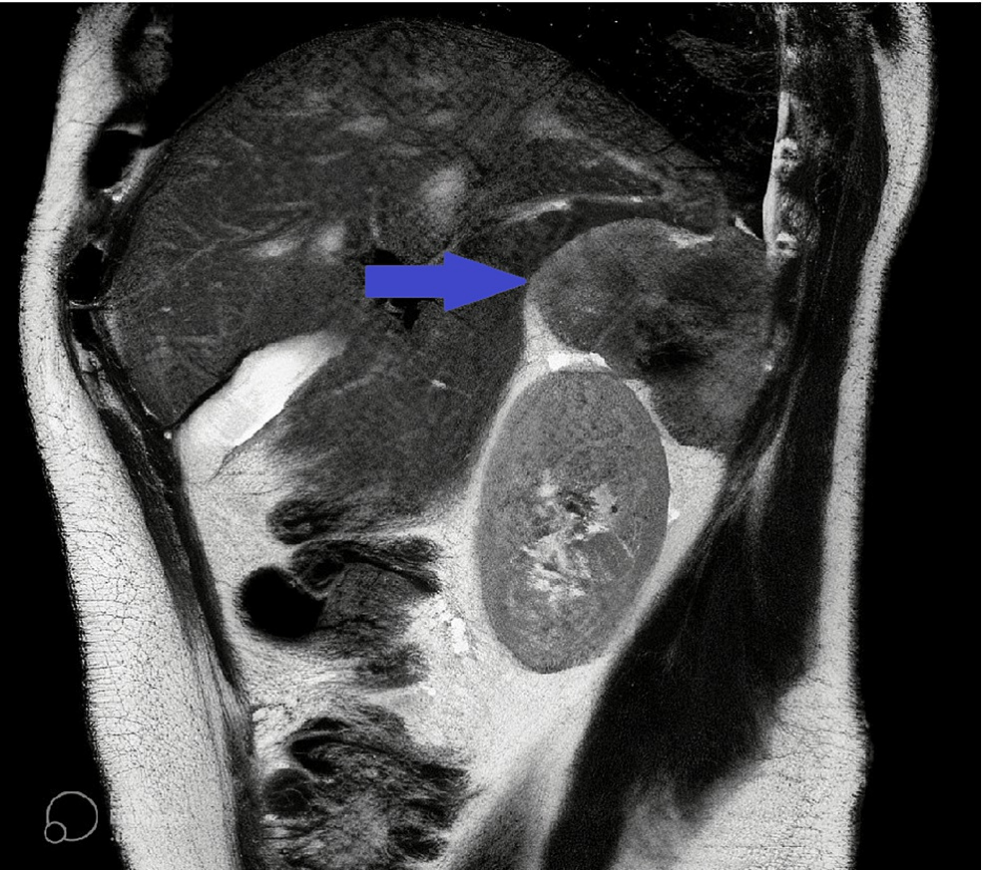
Magnetic resonance image of the retroperitoneal synovial sarcoma localized between the liver, diaphragm, and upper pole of the right kidney.

A 35-year-old male patient who underwent abdominal CT in an external center due to intermittent right flank pain lasting for four months was referred to our hospital following the detection of a central calcified mass in the retroperitoneum (Figure 1). He had no known comorbidity. It was determined that he had been working in a facility manufacturing automotive tires for the last four years. His physical examination was unremarkable, and the mass was non-palpable. All laboratory parameters were normal. Magnetic resonance imaging revealed a heterogeneous, contrast-enhanced, diffusion-limiting mass lesion measuring 83 × 53 × 63 mm in the retroperitoneal area adjacent to the upper pole of the right kidney and right adrenal gland with borders that could be distinguished from these organs but could not be distinguished from the diaphragm (Figure 2). No pathological formation was observed on thorax CT, and the patient was considered to have a primary non-metastatic RPM.

**Figure 3:**
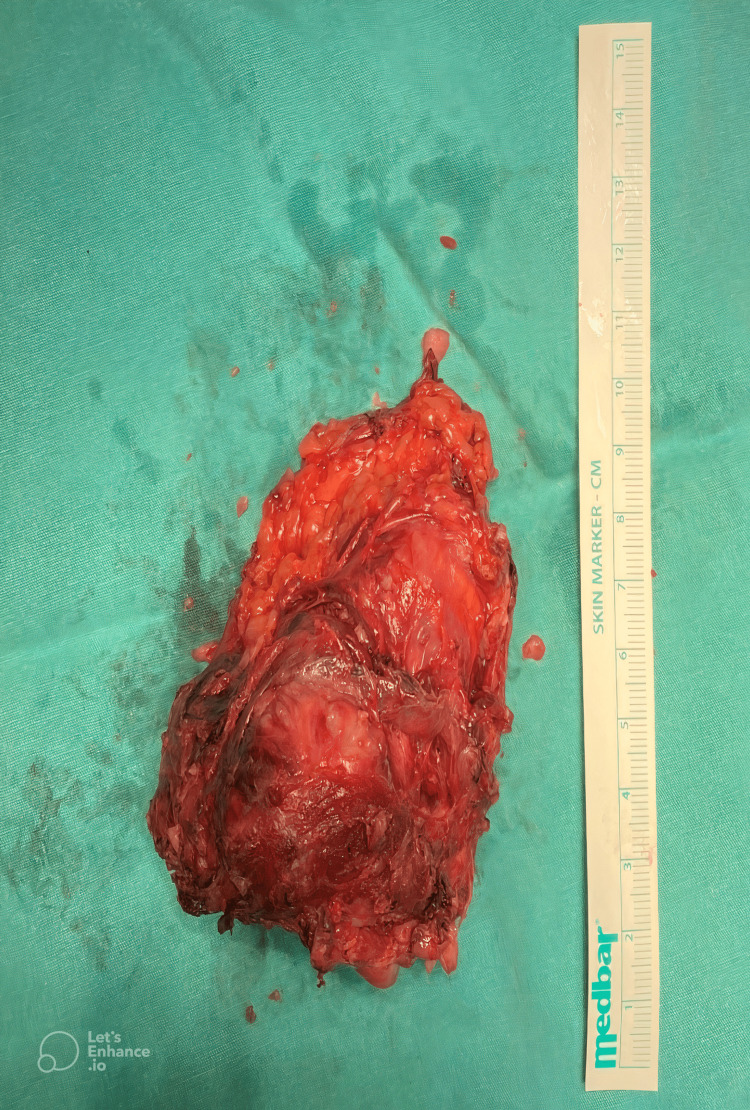
Photograph of the excised specimen.

It was decided to perform laparoscopic transperitoneal resection of the mass. The camera port was placed lateral to the rectus abdominis muscle at the level of the umbilicus, the second trocar was placed 10 cm lateral to the camera port at the level of the umbilicus, the third trocar was placed at the junction of the rectus abdominis muscle and the rib, and the fourth trocar, which was used for liver retraction, was placed under the xiphoid. It was observed that the mass was highly invading the right psoas muscle, and a wide resection was performed in this area together with the muscle tissue. Upon recognizing that the mass had completely invaded the diaphragm and could not be separated from this area, we removed approximately 5 cm of the diaphragm en bloc with the mass to not leave any tumor tissue behind (Figure 3). It was observed that the pleura was not opened, and, therefore, the open diaphragm was sutured primarily and the procedure was terminated laparoscopically. Operative time was 110 minutes and there was no significant bleeding. There were no complications in the postoperative period. The patient was discharged on the third postoperative day. The histopathological analysis (R0 complete resection) revealed a monophasic SS with prominent calcified/ossified areas and a Ki67 proliferative index of >10%, upon which adjuvant chemo/radiotherapy was planned. The patient received the DOXOIFOS regimen (doxorubicin and ifosfamide) but he refused radiotherapy and it was not applied. In the radiological and clinical evaluation performed 12 months postoperatively, there was no finding of local or systemic spread.

## Discussion

Although there is no certainty in the etiology of sarcomas, exposure to radiation and chemicals (e.g., polyvinyl chloride, thorotrast, chlorophenols, etc.) and genetic changes (translocations and fusion genes) have been implicated as causative factors. Almost all SSs contain translocation t(X,18), which is specific to this cancer type [5]; therefore, the detection of this translocation is of diagnostic importance. *SYT-SSX1* or *SYT-SSX2* fusion genes emerging from the fusion of the *SYT* gene on chromosome 18 (18q11) and the *SSX1* or *SSX2* gene on the X chromosome (Xp11) encode transcription-activating proteins [5]. These fusion proteins are considered to play a role in tumorigenesis. Some studies have shown that patients with SSs with the *SYT-SSX1* fusion gene have a worse prognosis in terms of overall survival than those with *SYT-SSX2* [6]. On the other hand, there are also studies reporting that the type of fusion does not alter the prognosis [5]. Although the effect of the type of translocation on the prognosis remains controversial, the positive effect of effective surgical treatment is indisputable. The most effective factor contributing to disease-free survival is a negative margin obtained as a result of the surgical resection of the tumor. For example, in a study investigating the survival outcomes of a series of 500 patients who underwent surgical treatment, the median survival was 103 months in patients with a negative margin versus 18 months in those who had undergone incomplete resection [1]. However, due to the proximity of the mass to vital structures, gross surgical resection with negative margins can only be obtained in fewer than 70% of the cases. In addition, some studies have shown that a significant portion of patients with RPSs who have undergone complete macroscopic resection may have local recurrence and systemic spread during follow-up. A study conducted by the French Sarcoma Group revealed that the five-year recurrence-free survival rate of patients who underwent complete macroscopic resection was 46%, and the five-year overall survival rate was 66% [7]. The same study reported that factors such as adjacent organ involvement, surgeon specialization, piecemeal resection, gender, and perioperative radiotherapy were associated with local recurrence [7]. Multimodal treatment (surgery with radiotherapy and/or chemotherapy) should be applied in patients with risk factors for recurrence [8]. If radiotherapy is planned, it should preferably be applied in the preoperative period if possible. It has been reported that preoperative radiotherapy can reduce possible tumor seeding during surgery, increase tumor resectability, and is associated with lower complication rates [9]. However, in clinical practice, preoperative radiotherapy is not always possible due to a biopsy not being performed for each patient. In these patients, adjuvant or intraoperative radiotherapy can also be considered as an alternative. According to the results of a study examining the effects of chemotherapy on prognosis in histological subgroups of RPSs, chemotherapy does not contribute to overall survival in patients with liposarcomas, leiomyosarcomas, fibrosarcomas, and angiosarcomas, but it has positive effects on overall survival in those with spindle cell sarcomas, giant cell sarcomas, and SSs [10].

## Conclusions

RPSs may sometimes present as a calcified mass. Although surgical dissection of these masses is difficult, it can also be performed laparoscopically. The main step in the treatment of RPSs is to perform a complete resection but this is not always possible. Unfortunately, local recurrence and systemic spread still occur in a significant portion of patients who have undergone complete resection. To reduce this possibility, multimodal treatment (surgery with radiotherapy and/or chemotherapy) should be applied in some sub-histological groups, such as SSs.
